# Artificial intelligence can overcome challenges in brachytherapy treatment planning

**DOI:** 10.1002/acm2.13504

**Published:** 2022-01-18

**Authors:** Xun Jia, J. Adam M. Cunha, Yi Rong

**Affiliations:** ^1^ Department of Radiation Oncology University of Texas‐Southwestern Dallas Texas USA; ^2^ Department of Radiation Oncology University of California San Francisco San Francisco California USA; ^3^ Department of Radiation Oncology Mayo Clinic Arizona Phoenix Arizona USA

## 1 INTRODUCTION

In the field of radiotherapy (RT) in recent years, there has never been any diminution in enthusiasm of adopting artificial intelligence (AI), in the form of deep learning and/or machine learning, mainly for external beam treatment applications. A PubMed search with the keywords “artificial intelligence” and “radiotherapy” would return thousands in publications, whereas if the keywords are changed to “artificial intelligence” and “brachytherapy,” results would end up in lower hundreds. The interest in applying AI toward the brachytherapy planning process, including applicator digitization, contouring, plan optimization, and so forth has never fully blossomed. The reasons might be mainly twofold: (1) AI requires large amount of uniform training data, while “brachytherapy” might be the antonym of “uniform” and “large data,” considering the treatment variations and case amount at each institution; (2) each treatment planning step is straightforward but poses unique challenges, considering the use of multi‐modality images, image artifacts from applicators, case‐specific dose distribution, and so forth. Taking a walk down the memory lane, brachytherapy treatment planning has been advancing slowly in general compared to external beam RT, going from standard pear‐shape planning not too long ago to MRI‐guided high‐risk clinical planning volume gold‐standard in the recent years. When considering devoting limited resources in further advancing brachytherapy, should we take such a big leap to AI or should we take a conservative route exploring more traditional computation methods? In other words, do we have the faith that AI can ultimately overcome those challenges in brachytherapy and provide better solutions? In this debate, Dr. Xun Jia argues for the proposition that “AI can overcome challenges in brachytherapy treatment planning,” while Dr. J. Adam M. Cunha argues against it.

Dr. Xun Jia is Professor and Associate Vice Chair of Medical Physics Research at the Department of Radiation Oncology, University of Texas Southwestern Medical Center (UTSW). He received his master's degree in applied mathematics in 2007 and Ph.D. degree in physics in 2009, both from the University of California Los Angeles. After his postdoctoral training in medical physics from the Department of Radiation Physics and Applied Sciences, University of California San Diego, he became a faculty in the same department in 2011. He moved to UTSW in 2013. Over the years, Dr. Jia has conducted productive research on cone beam CT reconstruction, GPU‐based Monte Carlo radiation transport simulation, deep learning for image processing and RT treatment planning, and development of a preclinical small animal radiation research platform. He has published ∼150 peer‐reviewed manuscripts. His research has been funded by NIH, the State of Texas, industrial, and charitable funding agencies. Dr. Jia currently serves as an Executive Editorial board member of Physics in Medicine and Biology. He is the recipient of John Laughlin Young Scientist Award of American Association of Physicists in Medicine in 2017.

Dr. J. Adam M. Cunha is a resident of the San Francisco Bay Area in Northern California. He is an Associate Professor in the Department of Radiation Oncology at the University of California, San Francisco. Dr. Cunha first started working with machine learning in 2002 where it was a crucial part of his Ph.D. thesis searching for rare sub‐atomic particles at the SLAC National Laboratory. Since transitioning into medical physics Dr. Cunha has dedicated his career to improving brachytherapy practice. He has focused his research on optimization and hardware development including robotic devices, electromagnetic tracking, 3D printing, and treatment planning algorithms.

## 2 OPENING STATEMENT

### 2.1 Xun Jia, PhD

Brachytherapy treatment planning generally encompasses steps of imaging, structure delineation, dose calculation, plan optimization, and so forth. The goal here is to accurately accomplish these steps in a timely fashion. In recent years, numerous studies and commercial products have demonstrated success in building AI models to solve various problems in these steps. For instance, AI‐based segmentation algorithms can accurately delineate the treatment target, organs at risk, and brachytherapy applicators and seeds for commonly used imaging modalities in brachytherapy, such as CT, MRI, and ultrasound.^1^, ^2^, ^3^ AI methods for dose calculations have also become available to enable calculations with tissue heterogeneity considered.^4^

Compared to external beam RT, there are unique challenges in brachytherapy. Yet it is conceivable that, with proper adjustments, AI tools built‐in external beam RT can be adapted to solve brachytherapy problems. Take the organ segmentation problem as an example, AI models can be refined to handle this problem on images with relatively poor quality caused by fast data acquisition in brachytherapy practice or artifacts generated by applicators or seeds. Meanwhile, many AI tools are built to address generic problems across multiple fields including brachytherapy. They are hence capable of solving problems in brachytherapy treatment planning. For example, AI‐based CT metal artifact reduction methods can mitigate imaging artifacts caused by metal objects. Material decomposition methods can provide valuable material compositions information to support accurate radiation dose calculation in the low energy range.

Modern CT‐ or MRI‐guided brachytherapy is often subject to an intense time pressure, requiring imaging, contouring, and planning all completed while the applicators are implanted in the patient. It is therefore vital to perform the planning tasks in a timely fashion. There have been several successes using AI and automations to streamline the planning process. The group at Sunnybrook cancer hospital built machine learning algorithms for low‐dose rate brachytherapy treatment planning of prostate cancer, yielding an average planning time of 0.84 min, compared with 17.88 min for expert planners,^5^ and subsequently demonstrated noninferior postoperative dosimetry achieved by the algorithm via a Phase I clinical trial.^6^ Having an AI model to digitize the large number of needle applicators in interstitial brachytherapy is another example of improving efficiency.^2^ Via reducing human interventions, automation helps mitigating potential human errors that can be manifested when time is pressed.

Needless to say, brachytherapy treatment planning often encounters complex situations that require human decisions, for example, to decide dosimetric tradeoffs in organs and/or targets. This aspect, namely building intelligence that can autonomously make decisions like humans do, is indeed at the core of modern AI, as indicated by the word “intelligence” in “AI.” In the past few years, we are fascinated to see that virtual game players can play complex computer games or board games like Go, and beat top human players.^7^ Recent advances using deep reinforcement learning, the backbone of these virtual game players, have enabled human‐like treatment planning in brachytherapy—a virtual planner operating a treatment planning system to derive high‐quality plans.^8^ I envision a future that human planners will partner and interact with virtual colleagues in brachytherapy treatment planning, with the AI colleagues working on the actual tasks and the humans mainly taking responsibility for quality review and making corrections if necessary. This AI‐based decision‐making capability is expected to be of particular importance for brachytherapy treatment planning in resource‐limited settings, with the hope to fill the shortage of human expertise due to insufficient staffing or training.

On the translation side, one challenge impeding clinical deployment of AI models is their robustness.^9^ Models with nonrobust behaviors necessitate planners carefully inspecting the results from the AI models and correcting errors, which obviously counteracts the whole benefit of using AI. This problem is often caused by different dataset characteristics used for model development and clinical application, due to, for example, different image acquisition protocols, resulted in nongeneralizable AI models. This is indeed not a unique problem to brachytherapy but generally a concern for all AI applications in medicine. With strong desires to achieve the best AI performance in real clinical practice, rapid advancements such as domain adaptation and adversarial training have substantially improved generalizability and robustness of AI models.

Learning and continuous learning are also core components of the AI framework, which critically supports clinical translation of AI models.^10^ In practice, each institution may have its own planning protocols. Specific guidelines in brachytherapy treatment planning, for example, dosimetric constraints, are also evolving with knowledge derived from new clinical studies. The learning capability of AI enables adaptations of models to specific clinical contexts, allowing them to self‐adjust to planning guidelines, to catch up with the state of the art, and to correct improper model behaviors.

In summary, AI has demonstrated its power in brachytherapy planning. New developments building intelligent models further empower the planning process with human expertise. Recent developments support the clinical translation of AI. With these considered, I believe AI can overcome challenges in brachytherapy treatment planning.

### 2.1 J. Adam M. Cunha, PhD

At first blush you may be inspired to wonder: AI has already started having an impact on so many aspects of treatment planning, how can this statement NOT be true?

I concede up front that the argument in favor of this statement absolutely is on the right side of history: AI will almost certainly contribute to better treatment of our brachytherapy patients. Indeed, I would have been happy arguing either side of this debate simply because who would argue against the progress of innovation! And yet, I have found composing this counter argument to be enlightening for two unique reasons. Each of these I hope spurs you to give your intuition pause: (1) pure computational power is a mighty tool and (2) brachytherapy planning relies heavily on externalities.

For decades, inverse planning optimization algorithms have been available that quickly output plans near the optimization Pareto Front. Inverse planning simulated annealing, for example, has been shown to reliably get to within a few percent of absolute optimality within a few seconds.^11^ Of course, this still requires feedback response as the planner fine tunes the solution for each patient. But even this limitation is solved by recent breakthroughs. Multi‐criteria optimization (MCO), for example, can probe the *entire* search space, generating tens of thousands of plans, in a matter of seconds.^12^ Upon commercial adoption, we will be able to peruse the optimization Pareto Front as easily as one scrolls through slices of a CT—notably, this is achievable simply by exploiting the continuing validity of Moore's Law via graphics processing unit (GPU)‐based computation. No planning‐specific AI is needed.

So, let us take a step back and identify what exactly are the challenges we face in brachytherapy treatment planning.

- Time: Treatment planning can be an onerous endeavor without the right tools.
- Consistency: The caliber of plan quality for a given set of contours needs consistency across various experience levels and toolset available to each planner.
- Accommodation: We need to be able to identify and accommodate unique patient‐specific restrictions on dose (internal injury, prior radiation, etc.).
- Robustness: Further automation will benefit from treatment plans that mitigate inevitable uncertainties like catheter migration and anatomical changes between imaging and delivery.
- Feasibility: Treatment planning requires that the implant is feasible, that is, the target is reachable via a brachytherapy applicator or catheters.
- Appropriateness: It is related to feasibility, but in the context of tumor biology or medical history: is brachytherapy the right treatment?
- Delineation: Identification of the target and avoidance structures has been shown to have a high enough variance that real and as‐delivered dosimetry can be impacted significantly depending on how contours were generated.

To evaluate the veracity of our thesis in each of these cases, it is helpful to define a razor that can be applied to each in turn. I propose the following:

Can this challenge be accomplished with brute force computing alone?

Is this challenge best solved in treatment planning or is an externality more fundamental?

In the spirit of Ockham's razor, the first component asks if there is already a simpler avenue to solve the challenge. While the second probes whether this challenge is most appropriately addressed at the treatment planning stage. Let us see what we get when we apply our razor to each challenge.

*Planning time* and *plan consistency* are fundamentally a product of our limited ability to probe the entire search space of our optimization problem. Once contours and the implant are digitized, the problem is defined completely. As the recent results with MCO have shown, we now have the computational power to quickly find every possible solution for a given implant and patient geometry. Thus, the first criterion of our razor is met: brute force computation alone can solve this challenge without the need for AI.

*Accommodation* requires thoughtful evaluation and understanding of the nuances of a specific case. At first glance, one might consider this a prime candidate for AI. But at the level of treatment planning, accommodation is mathematically a natural extension of plan consistency—we accommodate by imposing a reduced dose tolerance or weighing more heavily normal tissue sparing during optimization. Each of these can be represented as a term in our optimization algorithm. Thus, we reduce this problem to one of consistency: *q.e.d*. (If this is not adequate for your taste, I note that the nuanced decision making that does occur in accommodation happens prior to treatment planning. For example, what dose should we give to a patient who had prior RT? Following this logic, Accommodation is more appropriately considered an externality and satisfies our razor's second criterion.)

*Robust optimization*, like both plan consistency and accommodation, is fundamentally limited by our ability to compute all possible dosimetric outcomes due to changes to the implant‐anatomy geometry. Again, this is a computationally intensive problem; but it is one that is solvable in our current computing environment and therefore meets our razor's first query.

*Feasibility* and *appropriateness* are where things start to get interesting. Almost by definition, it is impossible to generate an acceptable brachytherapy plan if an implant cannot place the brachytherapy source near the tumor, that is, pubic arch interference blocking access to the prostate. Without proper applicator/seed implantation by an experienced brachytherapy expert, dose objectives will necessarily be unmet. Feasibility can be categorized as a subset of appropriateness: the selection of patients who, based on tumor biology, would benefit from brachytherapy (or the contraindication of patients unlikely to benefit) is critical. While feasibility and appropriateness both present a challenge for brachytherapy planning, the most apt solution is to determine who should get brachytherapy and who should not. Thus, when we apply our razor, these clearly fall under an externality. That is, while patient selection is one of the holy grails of AI in medicine, it is not fundamentally a treatment planning problem.

Finally, *delineation*, dear readers, is one challenge I must concede. Contouring is almost by definition personal and subject to nuance of image interpretation. The task is not well defined by traditional (non‐AI‐type) algorithms and therefore a solution is untenable by brute‐force computation. And delineation is so fundamentally tied to treatment planning, it would be disingenuous to categorize it as an externality. Therefore, we fail to meet either of our razor's criteria. This is not to say that AI can currently consider this problem solved. There remain all the issues that plague machine learning and deep learning across industries not least of which is the reliance on massive datasets, which will manifest differently at each institution.

In conclusion, I propose a more accurate statement: “AI does not need *to* solve many of the challenges in brachytherapy treatment planning.” Traditional computational methods that rely on brute force probing of the entirety of the possible solution set can tackle many of the challenges in brachytherapy treatment planning. AI is not needed so the original question is moot. However, contouring is one challenge that will very likely benefit from AI since this challenge requires statistical methods that interpret datasets (contours) that are fundamentally nondiscrete and irreducible.

## 3 REBUTTAL

### 3.1. Xun Jia, PhD

I agree with my opponent on the nicely summarized challenges in brachytherapy treatment planning and the spirit of Ockham's razor. But I would like to emphasize that brute force computation alone is not adequate, or not the most appropriate one, to address these challenges in many cases. For instance, it is true that we can mathematically formulate each planning objective, for example, dose tolerance. With high‐power computations, MCO enables searching the Pareto space, thereby potentially enhancing consistency and robustness. Nonetheless, this framework can only rapidly generate candidate plan solutions, but the time spent on browsing these solutions and decision making by a human planner is still needed and may potentially impede the goal of consistency and robustness considering various human factors such as training, experience, and available time in plan selection. In this regard, the intelligence aspect in AI, as explained in my opening statement, seamlessly complements MCO and closes the loop by behaving as a human planner for plan selection.

There are other components in the planning pipeline that are too complicated to be handled accurately and robustly by classical algorithms and may require human interventions. Applicator/seed digitization is a great example, especially in complex situations such as images with strong artifacts or cases with a large number of needles in intricate geometry configurations. Recent AI solutions have demonstrated successes in overcoming such challenges.

Further along this line, human‐like decision making is the holy grail of the AI world. Current AI studies mostly employed deep neural networks as a statistical tool for data mapping between different domains, for instance from CT images to needle configurations for needle digitization. Yet the ultimate goal of AI in medicine is indeed to develop trustworthy virtual partners to assist humans, also known as human‐centered AI. In a broad scope, I would consider challenges such as deciding the appropriate dose to a patient with prior radiation treatment, or determining a feasible and appropriate plan under sub‐optimal applicator placement, as part of brachytherapy treatment planning rather than its externalities. While no mature AI solutions at this moment, the pursuit for human‐centered AI will eventually empower us in better handling these challenges. Of note, I agree that properly placing applicators/seeds is the challenge that is apparently beyond the scope of planning. But one can not deny the fact that such problem will benefit from AI‐based image guidance tools.

Finally,, delineation, especially target delineation, is indisputability a challenge affecting more than just treatment planning, but the overall success of brachytherapy. There are two aspects in the delineation task: accuracy and consistency. Target delineation accuracy is normally proven by clinical outcome analysis. AI tools will be essential for this task, particularly for large‐scale analysis across multiple institutions with a modern federated learning scheme. Regarding consistency, AI solutions will help reducing variations among planners or institutions due to human subjectivity, experience level, guideline deviation, and so forth. More importantly, consistency improvement feeds back to the aspect of improving accuracy, by enhancing the overall quality of clinical outcome studies.

### 3.1 J. Adam M. Cunha, PhD

AI is here to stay. It certainly will be incorporated into many aspects of our practice. As Dr. Jia so aptly laid out in his argument, challenges that cannot be well defined, like contouring, will most certainly be met head on by AI. I have no doubt AI will help us do better, faster, and more consistent contouring for treatment planning. However, this is precisely the situation where it is difficult to ensure that the application of an algorithm is identical to the training used to develop the algorithm.

*Statistical learning (by definition) uses incomplete information to make an educated guess of the best answer*. This makes machine learning as subtle as it is powerful since it also begets its foil: it is only as good as its training. So, I would like to take the opportunity presented to me in this rebuttal to remind you, Dear Reader: Embrace AI, yes. But be aware. Poor training is dangerous because it can be very hard to identify, and it will mislead as often as not.

Except for structure delineation/contouring, brachytherapy treatment planning challenges can be simply expressed as solvable (though quite likely onerous) mathematical statements. Modern computational power continues to grow exponentially. This has enabled brute‐force probing of the search space for *the absolute best* solution by evaluating each and every possible solution within clinically meaningful tolerances, which is feasible and therefore must be favored over AI for the majority of the challenges in brachytherapy treatment planning. If the serach space is able to be completely probed in a reasonable time, this will *always* be more accurate than an answer obtained by inferring from statistical analysis.

In conclusion, we see the biggest gains from AI when applied to problems that have a multitude of both input and output parameters, or have loosely correlated relationships between inputs and outputs. This is true for contouring but is otherwise not a feature of the current challenges to brachytherapy treatment planning. In the cases where our challenges do push this boundary, however, current computing power allows us to find the most optimal solution using brute force evaluation of the entirety of the search space.

So, can AI overcome challenges in brachytherapy treatment planning? Possibly. But quite often so can simpler, more absolute methods.

## AUTHOR CONTRIBUTION

All authors contributed in topic conception and paper writing.
